# Synthesis, crystal structure and Raman spectrum of K_2_[(Pt_2_)(HPO_4_)_4_(H_2_O)_2_] containing (Pt_2_)^6+^ ions

**DOI:** 10.1107/S1600536809005601

**Published:** 2009-02-21

**Authors:** Kosta Panagiotidis, Robert Glaum

**Affiliations:** a Institut für Anorganische Chemie, Rheinische Friedrich-Wilhelms-Universität Bonn, Gerhard-Domagk-Strasse 1, D-53121 Bonn, Germany

## Abstract

In the crystal structure of the acid platinum phosphate dipotassium di-μ-hydrogenphosphato-bis­[aqua­platinum(III)](*Pt*—*Pt*), K_2_[Pt_2_(HPO_4_)_4_(H_2_O)_2_], the (Pt_2_)^6+^ dumbbells within the paddle-wheel complex show Pt—Pt distances of 2.4944 (5) and 2.4892 (5) Å. The pottassium ions are seven-fold coordinated by hydrogenphosphate groups. In the crystal, O—H⋯O hydrogen bonds help to establish the packing. The Raman spectrum was recorded.

## Related literature

The structure of the title isotypic sodium compound, Na_2_[Pt_2_(HPO_4_)_4_(H_2_O)_2_], was determined by Cotton *et al.* (1982*a* ▶). For platinum phosphates, see: Wellmann & Liebau (1981 ▶). For related compounds containing dinuclear plati­num(III), see: Bancroft *et al.* (1984 ▶); Baranovskii & Schelokow (1978 ▶); Che *et al.* (1982 ▶); Cotton & Walton (1982*b* ▶); Dikareva *et al.* (1982 ▶, 1987 ▶); Muraveiskaya *et al.* (1974 ▶, 1976 ▶); Pley & Wickleder (2004*a* ▶,*b* ▶, 2005 ▶); Stein *et al.* (1983 ▶); Woollins & Kelly (1985 ▶). For the ternary system Pd/P/O, see: Palkina *et al.* (1978 ▶); Panagiotidis *et al.* (2005 ▶). For hydrogen bonds, see: Steiner (2002 ▶). For the Raman spectra of In_3_(PO_4_)_2_ and In_2_O(PO_4_), see: Thauern & Glaum (2004 ▶). For the synthesis, see: Brauer (1978 ▶).

## Experimental

### 

#### Crystal data

K_2_[Pt_2_(HPO_4_)_4_(H_2_O)_2_]
*M* *_r_* = 888.32
Triclinic, 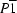

*a* = 7.8852 (2) Å
*b* = 7.9657 (2) Å
*c* = 13.7739 (4) Å
α = 82.358 (1)°
β = 81.509 (1)°
γ = 65.528 (1)°
*V* = 776.32 (4) Å^3^
*Z* = 2
Mo *K*α radiation
μ = 19.05 mm^−1^
*T* = 123 K
0.24 × 0.14 × 0.08 mm

#### Data collection

Nonius Kappa CCD diffractometer
Absorption correction: multi-scan (Blessing, 1995 ▶) *T*_min_ = 0.060, *T*_max_ = 0.224
18915 measured reflections
5589 independent reflections
4077 reflections with *I* > 2σ(*I*)
*R*_int_ = 0.064

#### Refinement

*R*[*F* ^2^ > 2σ(*F* ^2^)] = 0.033
*wR*(*F* ^2^) = 0.086
*S* = 0.98
5589 reflections
259 parameters
10 restraints
H atoms treated by a mixture of independent and constrained refinement
Δρ_max_ = 3.46 e Å^−3^
Δρ_min_ = −2.87 e Å^−3^

### 

Data collection: *COLLECT* (Hooft, 2004 ▶); cell refinement: *SCALEPACK* (Otwinowski & Minor, 1997 ▶); data reduction: *DENZO* (Otwinowski & Minor 1997 ▶) and *SCALEPACK*; program(s) used to solve structure: *SHELXS97* (Sheldrick, 2008 ▶); program(s) used to refine structure: *SHELXL97* (Sheldrick, 2008 ▶); molecular graphics: *ATOMS* (Dowty, 2006 ▶); software used to prepare material for publication: *WinGX* (Farrugia, 1999 ▶).

## Supplementary Material

Crystal structure: contains datablocks I, global. DOI: 10.1107/S1600536809005601/er2058sup1.cif

Structure factors: contains datablocks I. DOI: 10.1107/S1600536809005601/er2058Isup2.hkl

Additional supplementary materials:  crystallographic information; 3D view; checkCIF report

## Figures and Tables

**Table 1:** Hydrogen-bond geometry (Å, °)

*D*—H⋯*A*	*D*—H	H⋯*A*	*D*⋯*A*	*D*—H⋯*A*
O1—H1*A*⋯O31^i^	0.83 (6)	1.73 (6)	2.559 (7)	173 (8)
O1—H1*B*⋯O21^ii^	0.84 (6)	2.09 (4)	2.860 (7)	153 (8)
O14—H14⋯O31^iii^	0.83 (2)	1.82 (5)	2.548 (7)	146 (9)
O24—H24⋯O11^iv^	0.84 (7)	1.73 (7)	2.562 (7)	177 (9)
O34—H34⋯O41^v^	0.83 (6)	1.74 (3)	2.546 (6)	162 (9)
O10—H10*A*⋯O41^vi^	0.86 (2)	1.94 (4)	2.713 (7)	148 (7)
O10—H10*B*⋯O11^vii^	0.86 (2)	1.86 (2)	2.694 (6)	164 (6)
O42—H42⋯O21^viii^	0.84 (7)	2.27 (9)	2.475 (7)	94 (6)

Symmetry codes: (i) 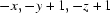; (ii) 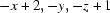; (iii) 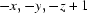; (iv) 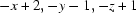; (v) 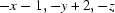; (vi) 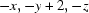; (vii) 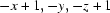; (viii) 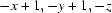.

**Fig. 1.**
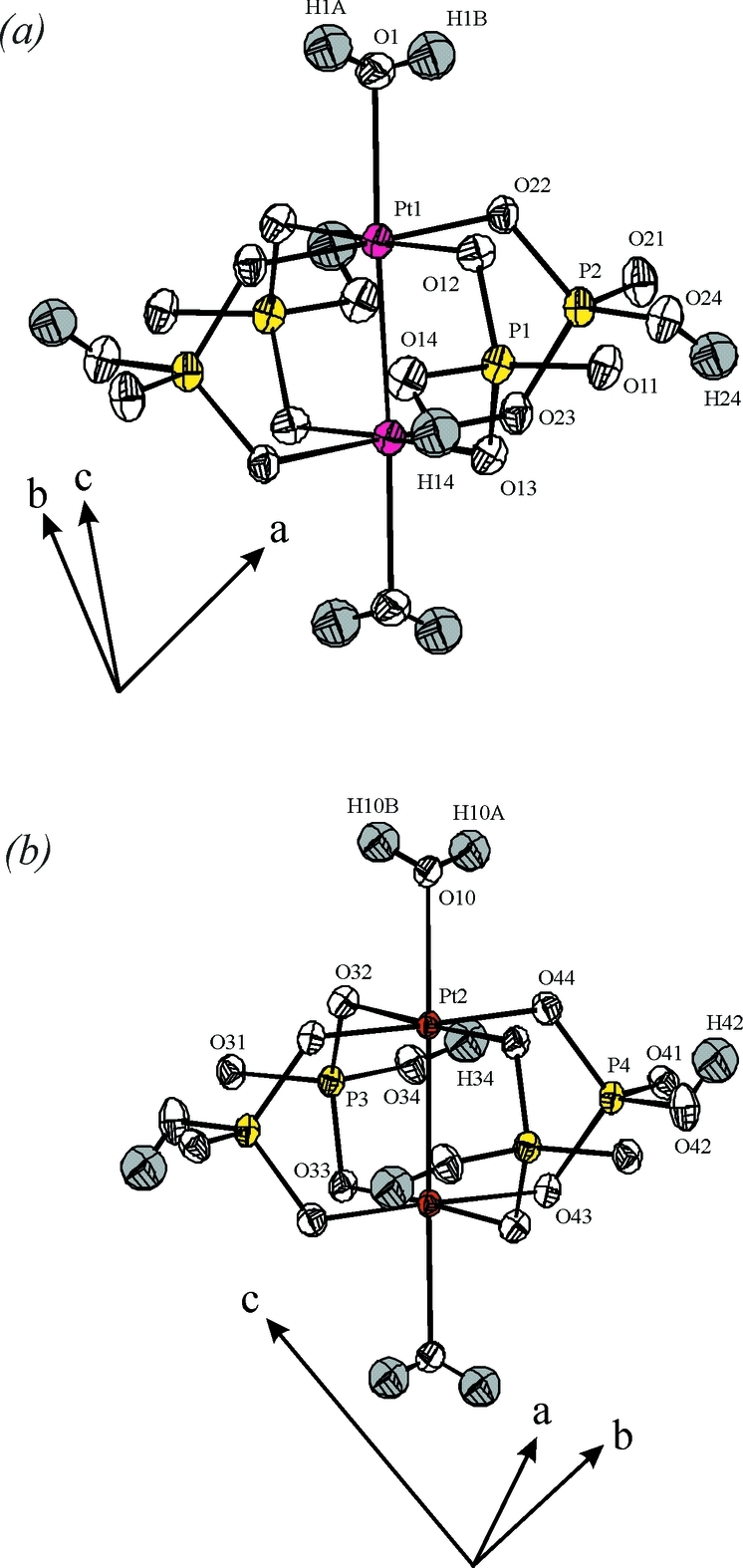
Pa ddle-wheel complex [(PtIII2)(HPO4)4(H2O)2] with anisotropic displacement parameters given at the 50% level (Progr. ATOMS v.6.2).

**Fig. 2.**
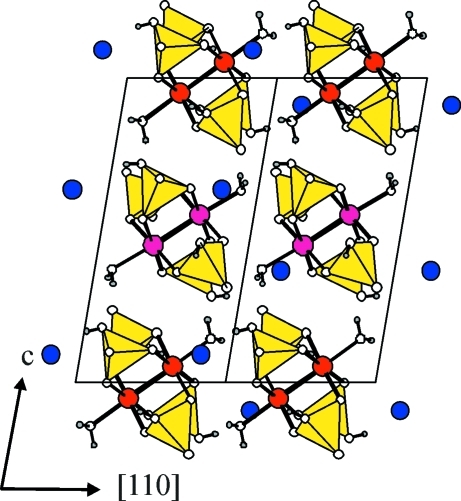
Projection of the crystal strucure of K2[(Pt2)(HPO4)4(H2O)2] along [110]. [PO4] tetrahedra (yellow), Pt26+ (red), K+ (violet), H+ (blue), O2- (white) (Progr. ATOMS v.6.2).

**Fig. 3.**
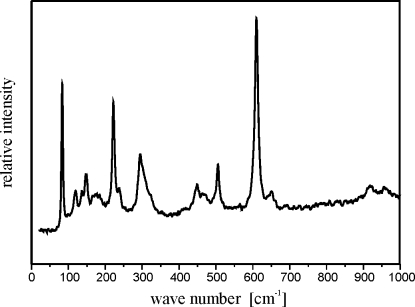
Raman spectrum of K2[(Pt2)(HPO4)4(H2O)2].

## supplementary crystallographic information

### Comment

Information on phosphates of noble metals like platinum and palladium is scarcely found in literature. During our recent investigation of the ternary system Pd/P/O we obtained the diphosphate Pd^II^_2_P_2_O_7_ (Panagiotidis *et al.*, 2005) in addition to the already known Pd(PO_3_)_2_ (Palkina *et al.*, 1978). In this context we were also interested in the crystal chemistry of platinum phosphates. Up to now, Pt^IV^P_2_O_7_ (Wellmann & Liebau, 1981) is the only structurally characterized anhydrous phosphate of platinum. Since reactions starting from "PtO×3H_2_O" and P_4_O_10_ did not yield products suitable for closer investigation we tried an alternative synthetic approach by reacting K_2_PtCl_4_ with conc. H_3_PO_4 _(see Experimental). This led to the formation of orange crystals of K_2_[(Pt_2_)(HPO_4_)_4_(H_2_O)_2_]. The acid phosphate is isotypic to the sodium compound (Cotton *et al.*, 1982*a*). In contrast to the structure of Na_2_[(Pt_2_)(HPO_4_)_4_(H_2_O)_2_] no disordered oxygen atoms are observed in the potassium compound. Distances d(Pt—Pt) for both structures are identical. The conventional residual as well as the standard deviations of the interatomic distances are slightly smaller for the refinement of K_2_[(Pt_2_)(HPO_4_)_4_(H_2_O)_2_]. The (Pt_2_)^6+^ binuclear complex was first observed in the crystal structure of K_2_[(Pt_2_(SO_4_)_4_(H_2_O)_2_] (Muraveiskaya *et al.*, 1974; Muraveiskaya *et al.*, 1976). By reaction of elemental platinum with concentrated sulfuric acid various platinum(III) sulfates were recently synthesized and structurally characterized (Pley & Wickleder, 2004*a*,b; Pley & Wickleder, 2005).

In K_2_[(Pt_2_)(HPO_4_)_4_(H_2_O)_2_] two crystallographically equivalent platinum atoms are connected to form (Pt_2_)^6+^ dinuclear complexes with surrounding (HPO_4_)^2-^ groups (Fig. 1). The ligating oxygen atoms of the (HPO_4_)^2-^ ions are arranged in a square-planar coordination around each platinum atom. Distances *d*(Pt—O) range from 1.978 Å to 2.030 Å. Angles 〈(O,Pt,*O*) are deviating only slightly from 90° and 180°, respectively. Due to their different crystal chemical environment, the oxygen atoms of the hydrogenphosphate anions show significantly different bond lenghts *d*(P—O). Oxygen atoms attached to platinum (coordination number of oxygen c.n.(O) = 2 (P, Pt)) show distances *d*(P—O) = 1.55 Å. Distances *d*(P—O) for those oxygen atoms which are coordinated to K^+^ ions (c.n.(O) = 2 (P, K)) range from 1.489 Å to 1.507 Å. Furthermore, for oxygen atoms which are attached to a hydrogen atom within the (HPO_4_)^2-^ unit (c.n.(O) = 2, (P, H)), distances *d*(P—OH) around 1.565 Å are observed. The axial ligand positions of the Pt_2_ dumbbells are occupied by water molecules (Fig. 1 & 2). Distances *d*(Pt—O) = 2.135 Å observed for the water ligands are significantly longer than those within the [Pt_2_O_8_] entity. The hydrogen atoms of [HPO_4_] tetrahedra (H14, H24, H34, H42; numbering according to the oxygen atoms that cary the hydrogen) and the water molecules (H1A, H1B, H10A, H10B) are involved in hydrogen bonding with oxygen atoms of adjacent [(Pt_2_)(HPO_4_)(H_2_O)_2_]^2-^ units. Interatomic distances d(OH···OP) range from 1.73 Å to 2.27 Å. They are in good accordance with those observed for strong hydrogen bonds (Steiner, 2002).

As found for various other compounds containing the (Pt_2_)^6+^ dinuclear complex, the angle 〈(Pt,Pt,*O*) between the dumbbell and the axial oxygen atoms deviates only slightly from 180° (Cotton *et al.*, 1982*b*; Pley & Wickleder *et al.*, 2005). [HPO_4_] tetrahedra in [(Pt_2_)(HPO_4_)(H_2_O)_2_]^2-^ show no significant angular distortion. Charge compensation of the anionic [Pt^III^_2_(HPO_4_)_4_(H_2_O)_2_]^2-^ unit is achieved by two crystallographically independent K^+^ ions, which are surrounded by oxygen atoms of phosphate groups.

In addition to its structure refinement, we were able to record the Raman spectrum of the paddle-wheel complex [Pt^III^_2_(HPO_4_)_4_(H_2_O)_2_]^2-^ (Fig. 3). An unequivocal assignment of the observed signals is yet impossible. Comparison of the Raman spectrum to those of the In_2_^4+^ containing indium phosphates In_3_(PO_4_)_2_ and In_2_O(PO_4_) (Thauern & Glaum, 2004) suggests the Pt—Pt valence vibration to be at ν = 222 cm^-1^. In comparison to ν(Pt—Pt) in complexes [(Pt^III^_2_)*L*_4_L'_2_]^n-^ (Stein *et al.*, 1983) assignment of ν = 83 cm^-1^ to the Pt—Pt vibration appears to be unreasonable. This is the more so, since *d *(Pt—Pt) = 2.51 Å observed for K_2_[(Pt_2_)(HPO_4_)_4_(H_2_O)_2_] is close to the lower limit of 2.47 Å < *d *(Pt—Pt) < 2.695 Å found for a series of dinuclear platinum(III) complexes (Che *et al.*, 1982, Stein *et al.*, 1983, Muraveiskaya *et al.*, 1974).

### Experimental

Aiming at the crystallization of "Pt_2_P_2_O_7_" a reaction starting from 150.0 mg K_2_Pt^II^Cl_4_, which were dissolved in water and mixed with 5.0 ml conc. H_3_PO_4_, was performed. After the obtained red solution was kept in a desiccator over P_2_O_5_ for two weeks, plate-like, orange crystals of K_2_[(Pt_2_)(HPO_4_)_4_(H_2_O)_2_] with edge-lengths up to 0.3 mm were deposited besides microcrystalline platinum (eq. 1). The synthesis of K_2_[Pt^II^Cl_4_] was performed according to the procedure given by Brauer (1978).

3 K_2_[Pt^II^Cl_4_] + 4 H_3_PO_4_ + 2 H_2_O → K_2_[(Pt^III^_2_)(HPO_4_)_4_(H_2_O)_2_] + Pt_s_ + 8H^+^ + 12 C l^-^ + 4 K^+^ (eq. 1)

### Crystal data

**Table:**

K_2_[Pt_2_(HPO_4_)_4_(H_2_O)_2_]	*Z* = 2
*M**_r_* = 888.32	*F*(000) = 812
Triclinic, *P*1	The lattice parameters of K_2_[Pt_2_(HPO_4_)_4_(H_2_O)_2_] were determined from single crystal diffraction data.
Hall symbol: -P 1	*D*_x_ = 3.800 Mg m^−^^3^
*a* = 7.8852 (2) Å	Mo *K*α radiation, λ = 0.71073 Å
*b* = 7.9657 (2) Å	Cell parameters from 5589 reflections
*c* = 13.7739 (4) Å	θ = 1.5–32.5°
α = 82.358 (1)°	µ = 19.05 mm^−^^1^
β = 81.509 (1)°	*T* = 123 K
γ = 65.528 (1)°	Plate, orange
*V* = 776.32 (4) Å^3^	0.24 × 0.14 × 0.08 mm

### Data collection

**Table:**

Nonius Kappa CCD diffractometer	5589 independent reflections
Radiation source: MoKα	4077 reflections with *I* > 2σ(*I*)
graphite	*R*_int_ = 0.064
Detector resolution: 9 pixels mm^-1^	θ_max_ = 32.5°, θ_min_ = 1.5°
CCD rotation images scans	*h* = −11→11
Absorption correction: multi-scan (Blessing, 1995)	*k* = −12→12
*T*_min_ = 0.060, *T*_max_ = 0.224	*l* = −19→20
18915 measured reflections	

### Refinement

**Table:**

Refinement on *F*^2^	Primary atom site location: structure-invariant direct methods
Least-squares matrix: full	Secondary atom site location: difference Fourier map
*R*[*F*^2^ > 2σ(*F*^2^)] = 0.033	Hydrogen site location: inferred from neighbouring sites
*wR*(*F*^2^) = 0.086	H atoms treated by a mixture of independent and constrained refinement
*S* = 0.98	*w* = 1/[σ^2^(*F*_o_^2^) + (0.0423*P*)^2^] where *P* = (*F*_o_^2^ + 2*F*_c_^2^)/3
5589 reflections	(Δ/σ)_max_ = 0.001
259 parameters	Δρ_max_ = 3.46 e Å^−^^3^
10 restraints	Δρ_min_ = −2.87 e Å^−^^3^
0 constraints	

### Special details

**Table:**

Geometry. All e.s.d.'s are estimated using the full covariance matrix. The cell e.s.d.'s are taken into account individually in the estimation of e.s.d.'s in distances and angles; correlations between e.s.d.'s in cell parameters are only used when they are defined by crystal symmetry. An approximate (isotropic) treatment of cell e.s.d.'s is used for estimating e.s.d.'s involving l.s. planes.
Refinement. Refinement of *F*^2^ against ALL reflections. The weighted *R*-factor *wR* and goodness of fit *S* are based on *F*^2^, conventional *R*-factors *R* are based on *F*, with *F* set to zero for negative *F*^2^. The threshold expression of *F*^2^ > σ(*F*^2^) is used only for calculating *R*-factors(gt) *etc*. and is not relevant to the choice of reflections for refinement. *R*-factors based on *F*^2^ are statistically about twice as large as those based on *F*, and *R*- factors based on ALL data will be even larger.

### Fractional atomic coordinates and isotropic or equivalent isotropic displacement parameters (Å^2^)

**Table:**

	*x*	*y*	*z*	*U*_iso_*/*U*_eq_	
K1	0.1242 (4)	−0.3657 (4)	0.6326 (2)	0.0790 (9)	
K2	0.3845 (3)	−0.1884 (3)	0.90856 (15)	0.0472 (5)	
O1	0.6393 (7)	0.2464 (8)	0.6286 (4)	0.0289 (11)	
H1A	0.566 (8)	0.299 (10)	0.676 (4)	0.043*	
H1B	0.734 (6)	0.175 (9)	0.656 (5)	0.043*	
O12	0.6784 (7)	−0.1304 (6)	0.6372 (3)	0.0238 (10)	
O14	0.4660 (7)	−0.2761 (7)	0.7170 (4)	0.0305 (11)	
H14	0.486 (13)	−0.387 (4)	0.717 (6)	0.046*	
O11	0.8028 (7)	−0.4742 (7)	0.6678 (3)	0.0256 (10)	
O13	0.5849 (7)	−0.3216 (6)	0.5370 (3)	0.0245 (10)	
O22	0.7832 (6)	0.0412 (7)	0.4628 (4)	0.0247 (10)	
O24	0.9754 (7)	−0.3003 (7)	0.4569 (3)	0.0276 (11)	
H24	1.045 (10)	−0.372 (10)	0.415 (5)	0.041*	
O21	0.9903 (7)	−0.0893 (7)	0.3117 (4)	0.0298 (11)	
O23	0.6936 (6)	−0.1446 (7)	0.3608 (3)	0.0267 (10)	
O10	0.2526 (7)	0.5993 (7)	0.1437 (3)	0.0249 (10)	
H10A	0.207 (11)	0.719 (3)	0.141 (5)	0.037*	
H10B	0.226 (11)	0.582 (9)	0.2058 (19)	0.037*	
O32	−0.1067 (6)	0.5907 (6)	0.1622 (3)	0.0212 (9)	
O33	−0.3103 (6)	0.5226 (6)	0.0567 (3)	0.0199 (9)	
O34	−0.4078 (7)	0.8408 (7)	0.1168 (4)	0.0286 (11)	
H34	−0.520 (4)	0.896 (11)	0.107 (6)	0.043*	
O31	−0.4038 (6)	0.5674 (7)	0.2362 (3)	0.0231 (10)	
O44	−0.0110 (6)	0.8018 (7)	0.0001 (3)	0.0236 (10)	
O41	−0.2456 (6)	1.0558 (6)	−0.0996 (3)	0.0233 (10)	
O43	−0.2059 (6)	0.7307 (6)	−0.1068 (3)	0.0210 (9)	
O42	0.0530 (7)	0.8330 (7)	−0.1816 (4)	0.0310 (12)	
H42	0.050 (13)	0.930 (7)	−0.162 (6)	0.047*	
P1	0.6381 (2)	−0.3051 (2)	0.63814 (12)	0.0220 (3)	
P2	0.8592 (2)	−0.1187 (2)	0.39488 (12)	0.0215 (3)	
P3	−0.3082 (2)	0.6280 (2)	0.14389 (12)	0.0180 (3)	
P4	−0.1056 (2)	0.8585 (2)	−0.09741 (12)	0.0187 (3)	
Pt1	0.54549 (3)	0.09217 (3)	0.549475 (17)	0.01966 (7)	
Pt2	0.09528 (3)	0.53533 (3)	0.052197 (16)	0.01543 (6)	

### Atomic displacement parameters (Å^2^)

**Table:**

	*U*^11^	*U*^22^	*U*^33^	*U*^12^	*U*^13^	*U*^23^
K1	0.101 (2)	0.087 (2)	0.0691 (17)	−0.0526 (18)	−0.0488 (16)	0.0208 (15)
K2	0.0525 (12)	0.0601 (13)	0.0406 (10)	−0.0346 (11)	0.0048 (9)	−0.0133 (9)
O1	0.030 (3)	0.030 (3)	0.029 (3)	−0.013 (2)	0.000 (2)	−0.010 (2)
O12	0.030 (2)	0.020 (2)	0.021 (2)	−0.011 (2)	−0.0040 (19)	0.0035 (18)
O14	0.032 (3)	0.028 (3)	0.028 (3)	−0.014 (2)	0.007 (2)	0.001 (2)
O11	0.028 (2)	0.021 (2)	0.019 (2)	−0.003 (2)	−0.0023 (19)	0.0031 (18)
O13	0.029 (2)	0.019 (2)	0.021 (2)	−0.006 (2)	−0.0018 (19)	0.0019 (18)
O22	0.019 (2)	0.023 (2)	0.030 (3)	−0.007 (2)	0.0021 (19)	−0.005 (2)
O24	0.029 (3)	0.026 (3)	0.019 (2)	−0.005 (2)	0.0028 (19)	0.0021 (19)
O21	0.022 (2)	0.031 (3)	0.029 (3)	−0.008 (2)	0.0055 (19)	0.007 (2)
O23	0.021 (2)	0.028 (3)	0.025 (2)	−0.005 (2)	0.0031 (19)	−0.006 (2)
O10	0.028 (2)	0.032 (3)	0.020 (2)	−0.018 (2)	−0.0068 (19)	0.0045 (19)
O32	0.018 (2)	0.023 (2)	0.020 (2)	−0.0061 (19)	−0.0014 (17)	−0.0005 (18)
O33	0.018 (2)	0.025 (2)	0.016 (2)	−0.0101 (19)	0.0043 (16)	−0.0019 (17)
O34	0.020 (2)	0.025 (3)	0.038 (3)	−0.006 (2)	−0.003 (2)	−0.001 (2)
O31	0.024 (2)	0.026 (3)	0.019 (2)	−0.013 (2)	0.0055 (18)	−0.0032 (18)
O44	0.021 (2)	0.023 (2)	0.028 (2)	−0.011 (2)	−0.0080 (18)	0.0054 (19)
O41	0.021 (2)	0.017 (2)	0.031 (3)	−0.0073 (19)	−0.0051 (19)	0.0016 (19)
O43	0.022 (2)	0.020 (2)	0.019 (2)	−0.0077 (19)	−0.0011 (17)	0.0025 (17)
O42	0.025 (2)	0.025 (3)	0.034 (3)	−0.007 (2)	0.008 (2)	0.004 (2)
P1	0.0237 (8)	0.0200 (8)	0.0183 (8)	−0.0061 (7)	−0.0002 (6)	0.0009 (6)
P2	0.0194 (8)	0.0218 (9)	0.0189 (8)	−0.0066 (7)	0.0020 (6)	0.0024 (6)
P3	0.0150 (7)	0.0204 (8)	0.0172 (7)	−0.0071 (6)	0.0016 (6)	−0.0005 (6)
P4	0.0167 (7)	0.0176 (8)	0.0190 (7)	−0.0057 (6)	−0.0011 (6)	0.0033 (6)
Pt1	0.02042 (12)	0.01997 (13)	0.01736 (12)	−0.00806 (10)	0.00056 (9)	−0.00042 (9)
Pt2	0.01457 (11)	0.01729 (12)	0.01430 (11)	−0.00707 (9)	−0.00109 (8)	0.00101 (8)

### Geometric parameters (Å, °)

**Table:**

K1—O24^i^	2.739 (6)	O24—P2	1.570 (5)
K1—O31^ii^	2.860 (5)	O21—P2	1.489 (5)
K1—O11^i^	2.957 (6)	O23—P2	1.549 (5)
K1—O42^iii^	3.047 (6)	O23—Pt1^iv^	2.014 (5)
K1—O22^iv^	3.053 (5)	O10—Pt2	2.132 (5)
K1—O32^ii^	3.156 (5)	O32—P3	1.544 (5)
K1—O12^i^	3.222 (6)	O32—Pt2	1.978 (4)
K2—O14	2.735 (6)	O33—P3	1.561 (5)
K2—O34^v^	2.822 (6)	O33—Pt2^vii^	2.030 (4)
K2—O41^v^	2.858 (5)	O34—P3	1.564 (5)
K2—O33^ii^	2.920 (5)	O31—P3	1.507 (4)
K2—O42^iii^	2.990 (6)	O44—P4	1.555 (5)
K2—O43^vi^	3.000 (5)	O44—Pt2	2.005 (5)
K2—O44^iii^	3.215 (5)	O41—P4	1.500 (5)
O1—Pt1	2.143 (5)	O43—P4	1.553 (5)
O12—P1	1.549 (5)	O43—Pt2^vii^	2.014 (4)
O12—Pt1	1.992 (4)	O42—P4	1.541 (5)
O14—P1	1.564 (5)	Pt1—O13^iv^	2.010 (4)
O11—P1	1.493 (5)	Pt1—O23^iv^	2.014 (5)
O13—P1	1.549 (5)	Pt1—Pt1^iv^	2.4944 (5)
O13—Pt1^iv^	2.010 (4)	Pt2—O43^vii^	2.014 (4)
O22—P2	1.541 (5)	Pt2—O33^vii^	2.030 (4)
O22—Pt1	1.985 (4)	Pt2—Pt2^vii^	2.4892 (5)
			
O11—P1—O12	110.2 (3)	O22—Pt1—O23^iv^	179.02 (19)
O11—P1—O13	110.9 (3)	O12—Pt1—O23^iv^	90.37 (19)
O12—P1—O13	111.5 (3)	O13^iv^—Pt1—O23^iv^	89.97 (19)
O11—P1—O14	110.2 (3)	O22—Pt1—O1	85.5 (2)
O12—P1—O14	104.8 (3)	O12—Pt1—O1	88.0 (2)
O13—P1—O14	109.0 (3)	O13^iv^—Pt1—O1	90.0 (2)
O21—P2—O22	111.1 (3)	O23^iv^—Pt1—O1	93.6 (2)
O21—P2—O23	113.2 (3)	O22—Pt1—Pt1^iv^	90.98 (14)
O22—P2—O23	109.6 (3)	O12—Pt1—Pt1^iv^	91.86 (14)
O21—P2—O24	106.9 (3)	O13^iv^—Pt1—Pt1^iv^	90.05 (14)
O22—P2—O24	107.7 (3)	O23^iv^—Pt1—Pt1^iv^	89.91 (15)
O23—P2—O24	108.1 (3)	O1—Pt1—Pt1^iv^	176.49 (14)
O31—P3—O32	108.9 (3)	O32—Pt2—O44	90.05 (19)
O31—P3—O33	109.3 (3)	O32—Pt2—O43^vii^	89.88 (18)
O32—P3—O33	111.8 (3)	O44—Pt2—O43^vii^	178.49 (18)
O31—P3—O34	111.6 (3)	O32—Pt2—O33^vii^	177.57 (17)
O32—P3—O34	106.3 (3)	O44—Pt2—O33^vii^	90.65 (19)
O33—P3—O34	108.9 (3)	O43^vii^—Pt2—O33^vii^	89.36 (18)
O41—P4—O42	110.7 (3)	O32—Pt2—O10	87.17 (19)
O41—P4—O43	109.3 (3)	O44—Pt2—O10	89.05 (19)
O42—P4—O43	110.0 (3)	O43^vii^—Pt2—O10	89.45 (19)
O41—P4—O44	110.6 (3)	O33^vii^—Pt2—O10	90.51 (18)
O42—P4—O44	106.4 (3)	O32—Pt2—Pt2^vii^	91.55 (13)
O43—P4—O44	109.7 (2)	O44—Pt2—Pt2^vii^	90.52 (13)
O22—Pt1—O12	89.21 (19)	O43^vii^—Pt2—Pt2^vii^	90.99 (13)
O22—Pt1—O13^iv^	90.42 (19)	O33^vii^—Pt2—Pt2^vii^	90.77 (13)
O12—Pt1—O13^iv^	178.06 (19)	O10—Pt2—Pt2^vii^	178.65 (14)

Symmetry codes: (i) *x*−1, *y*, *z*; (ii) −*x*, −*y*, −*z*+1; (iii) *x*, *y*−1, *z*+1; (iv) −*x*+1, −*y*, −*z*+1; (v) −*x*, −*y*+1, −*z*+1; (vi) *x*+1, *y*−1, *z*+1; (vii) −*x*, −*y*+1, −*z*.

### Hydrogen-bond geometry (Å, °)

**Table:**

*D*—H···*A*	*D*—H	H···*A*	*D*···*A*	*D*—H···*A*
O1—H1A···O31^v^	0.83 (6)	1.73 (6)	2.559 (7)	173 (8)
O1—H1B···O21^viii^	0.84 (6)	2.09 (4)	2.860 (7)	153 (8)
O14—H14···O31^ii^	0.83 (2)	1.82 (5)	2.548 (7)	146 (9)
O24—H24···O11^ix^	0.84 (7)	1.73 (7)	2.562 (7)	177 (9)
O34—H34···O41^x^	0.83 (6)	1.74 (3)	2.546 (6)	162 (9)
O10—H10A···O41^xi^	0.86 (2)	1.94 (4)	2.713 (7)	148 (7)
O10—H10B···O11^iv^	0.86 (2)	1.86 (2)	2.694 (6)	164 (6)
O42—H42···O21^xii^	0.84 (7)	2.27 (9)	2.475 (7)	94 (6)

Symmetry codes: (v) −*x*, −*y*+1, −*z*+1; (viii) −*x*+2, −*y*, −*z*+1; (ii) −*x*, −*y*, −*z*+1; (ix) −*x*+2, −*y*−1, −*z*+1; (x) −*x*−1, −*y*+2, −*z*; (xi) −*x*, −*y*+2, −*z*; (iv) −*x*+1, −*y*, −*z*+1; (xii) −*x*+1, −*y*+1, −*z*.
